# Urinary Collagen Fragments Are Significantly Altered in Diabetes: A Link to Pathophysiology

**DOI:** 10.1371/journal.pone.0013051

**Published:** 2010-09-28

**Authors:** David M. Maahs, Justyna Siwy, Àngel Argilés, Marie Cerna, Christian Delles, Anna F. Dominiczak, Nathalie Gayrard, Alexander Iphöfer, Lothar Jänsch, George Jerums, Karel Medek, Harald Mischak, Gerjan J. Navis, Johannes M. Roob, Kasper Rossing, Peter Rossing, Ivan Rychlík, Eric Schiffer, Roland E. Schmieder, Thomas C. Wascher, Brigitte M. Winklhofer-Roob, Lukas U. Zimmerli, Petra Zürbig, Janet K. Snell-Bergeon

**Affiliations:** 1 Barbara Davis Center for Childhood Diabetes, University of Colorado Denver, Aurora, Colorado, United States of America; 2 Mosaiques Diagnostics GmbH, Hannover, Germany; 3 RD – Néphrologie, Montpellier, France; 4 Department of General Biology and Genetics, Third Faculty of Medicine, Charles University, Prague, Czech Republic; 5 BHF Glasgow Cardiovascular Research Centre, University of Glasgow, Glasgow, United Kingdom; 6 Department of Cell Biology, Helmholtz Centre for Infection Research, Braunschweig, Germany; 7 Department of Medicine, University of Melbourne, Austin Health, Heidelberg, Victoria, Australia; 8 Division of Nephrology, Department of Internal Medicine, University Medical Centre Groningen, University of Groningen, Groningen, The Netherlands; 9 Division of Clinical Nephrology, Department of Internal Medicine, Medical University, Graz, Austria; 10 Steno Diabetes Center, Gentofte, Denmark; 11 Department of Internal Medicine, Third Faculty of Medicine, Charles University, Prague, Czech Republic; 12 Department of Nephrology and Hypertension, University of Erlangen-Nürnberg, Erlangen, Germany; 13 Metabolism and Vascular Biology Research Group, Department of Internal Medicine, Medical University of Graz, Graz, Austria; 14 Human Nutrition and Metabolism Research Center (HNMRC), Institute of Molecular Biosciences, Karl-Franzens University of Graz, Graz, Austria; University of Padova, Medical School, Italy

## Abstract

**Background:**

The pathogenesis of diabetes mellitus (DM) is variable, comprising different inflammatory and immune responses. Proteome analysis holds the promise of delivering insight into the pathophysiological changes associated with diabetes. Recently, we identified and validated urinary proteomics biomarkers for diabetes. Based on these initial findings, we aimed to further validate urinary proteomics biomarkers specific for diabetes in general, and particularity associated with either type 1 (T1D) or type 2 diabetes (T2D).

**Methodology/Principal Findings:**

Therefore, the low-molecular-weight urinary proteome of 902 subjects from 10 different centers, 315 controls and 587 patients with T1D (n = 299) or T2D (n = 288), was analyzed using capillary-electrophoresis mass-spectrometry. The 261 urinary biomarkers (100 were sequenced) previously discovered in 205 subjects were validated in an additional 697 subjects to distinguish DM subjects (n = 382) from control subjects (n = 315) with 94% (95% CI: 92–95) accuracy in this study. To identify biomarkers that differentiate T1D from T2D, a subset of normoalbuminuric patients with T1D (n = 68) and T2D (n = 42) was employed, enabling identification of 131 biomarker candidates (40 were sequenced) differentially regulated between T1D and T2D. These biomarkers distinguished T1D from T2D in an independent validation set of normoalbuminuric patients (n = 108) with 88% (95% CI: 81–94%) accuracy, and in patients with impaired renal function (n = 369) with 85% (95% CI: 81–88%) accuracy. Specific collagen fragments were associated with diabetes and type of diabetes indicating changes in collagen turnover and extracellular matrix as one hallmark of the molecular pathophysiology of diabetes. Additional biomarkers including inflammatory processes and pro-thrombotic alterations were observed.

**Conclusions/Significance:**

These findings, based on the largest proteomic study performed to date on subjects with DM, validate the previously described biomarkers for DM, and pinpoint differences in the urinary proteome of T1D and T2D, indicating significant differences in extracellular matrix remodeling.

## Introduction

Diabetes mellitus (DM) is a complex disease characterized by insufficient insulin production and resultant hyperglycemia with alterations in fat and protein metabolism. With time these alterations cause secondary cellular dysfunctions and vascular damage including diabetic nephropathy, retinopathy, neuropathy, and macrovascular disease or vascular alterations. The most common types of DM are type 1 diabetes (T1D) and type 2 diabetes (T2D). T1D is associated with destruction of insulin-producing β-cells in the islets of Langerhans in the pancreas, typically by an autoimmune mechanism, leading to insufficient insulin production. In contrast, T2D is caused by insulin resistance combined with insufficient insulin synthesis and is often associated with obesity.

Although all forms of DM are characterized by hyperglycemia and β-cell dysfunction, the pathogenesis of DM is variable, comprising different degrees of β-cell dysfunction, apoptosis, inflammation and immune responses. Proteome analysis holds the promise of delivering substantial insight into the pathophysiological changes associated with different types of DM. Urine represents an excellent specimen for proteome analysis, as it can be obtained in high quantities without the need for special collection procedures [1], shows higher stability than blood [2], [3], and enables the identification of valid biomarkers for renal, as well as systemic diseases [4], [5]. Recently, we identified and validated urinary proteomics biomarkers for DM, and DM associated micro- and macrovascular complications [3], [6]–[11]. These biomarkers also gave indications of relevant pathophysiological changes: the interference with homeostasis of extracellular matrix (ECM) turnover [9].

Based on these initial findings, we aimed to further validate urinary proteomics biomarkers for DM in general, and examine specific association of urinary proteins and peptides with either T1D or T2D. The identification of these differences in the urinary proteome should provide a deeper understanding of the pathophysiological changes associated with DM, especially DM associated micro- and macrovascular disease, and may result in advancements in therapeutic strategies.

## Results

### A. Urinary biomarkers for DM

**Figure 1:**
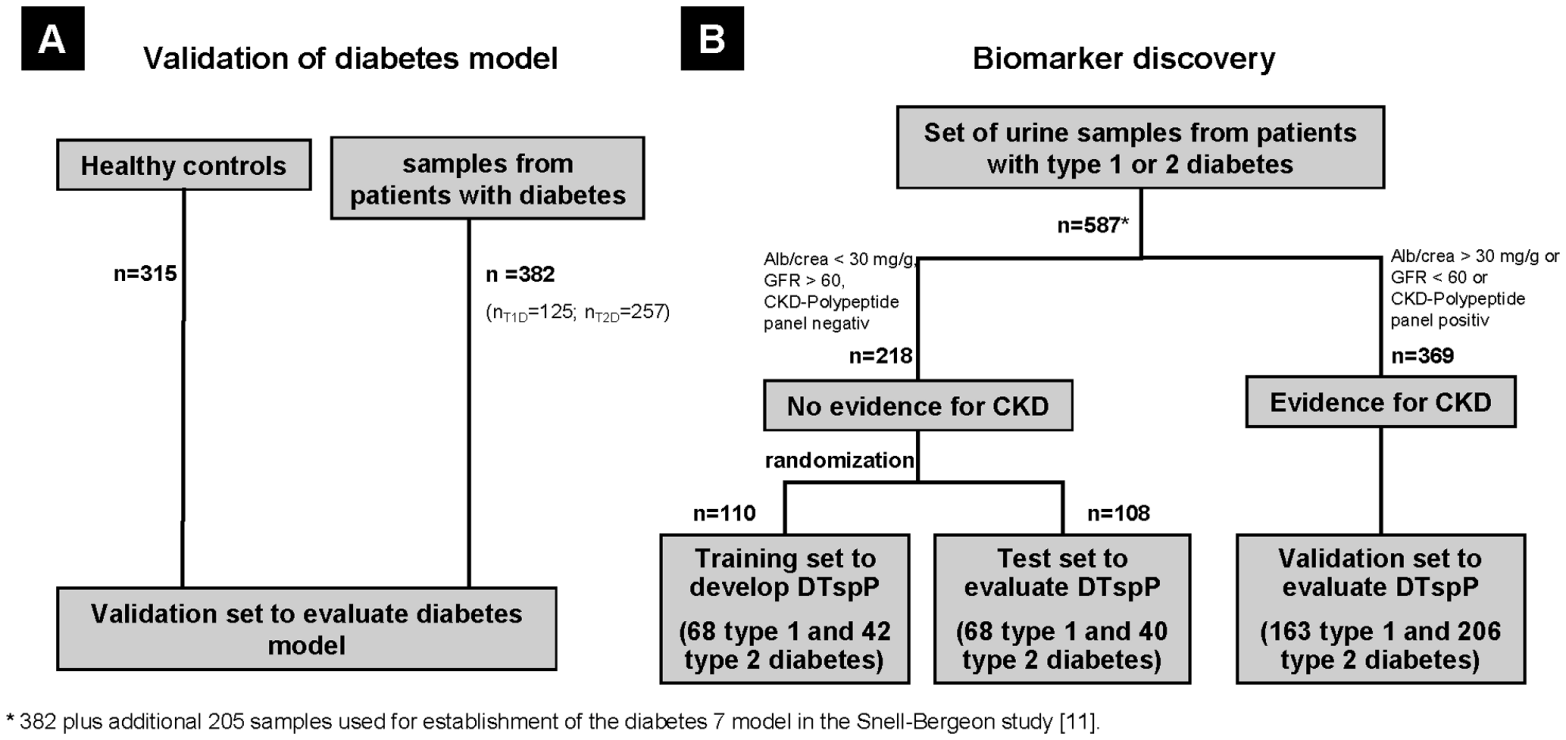
Study design. Flow chart describing the selection of samples used in this study. A: Urine samples from 697 individuals were analysed blinded, those contained 315 apparently healthy controls, and 382 urine samples from diabetic individuals. B: Samples from 587 well-characterized DM patients were used to identify DM type specific biomarkers. 382/587 samples were used for validation of previously described markers for DM.

**Figure 2:**
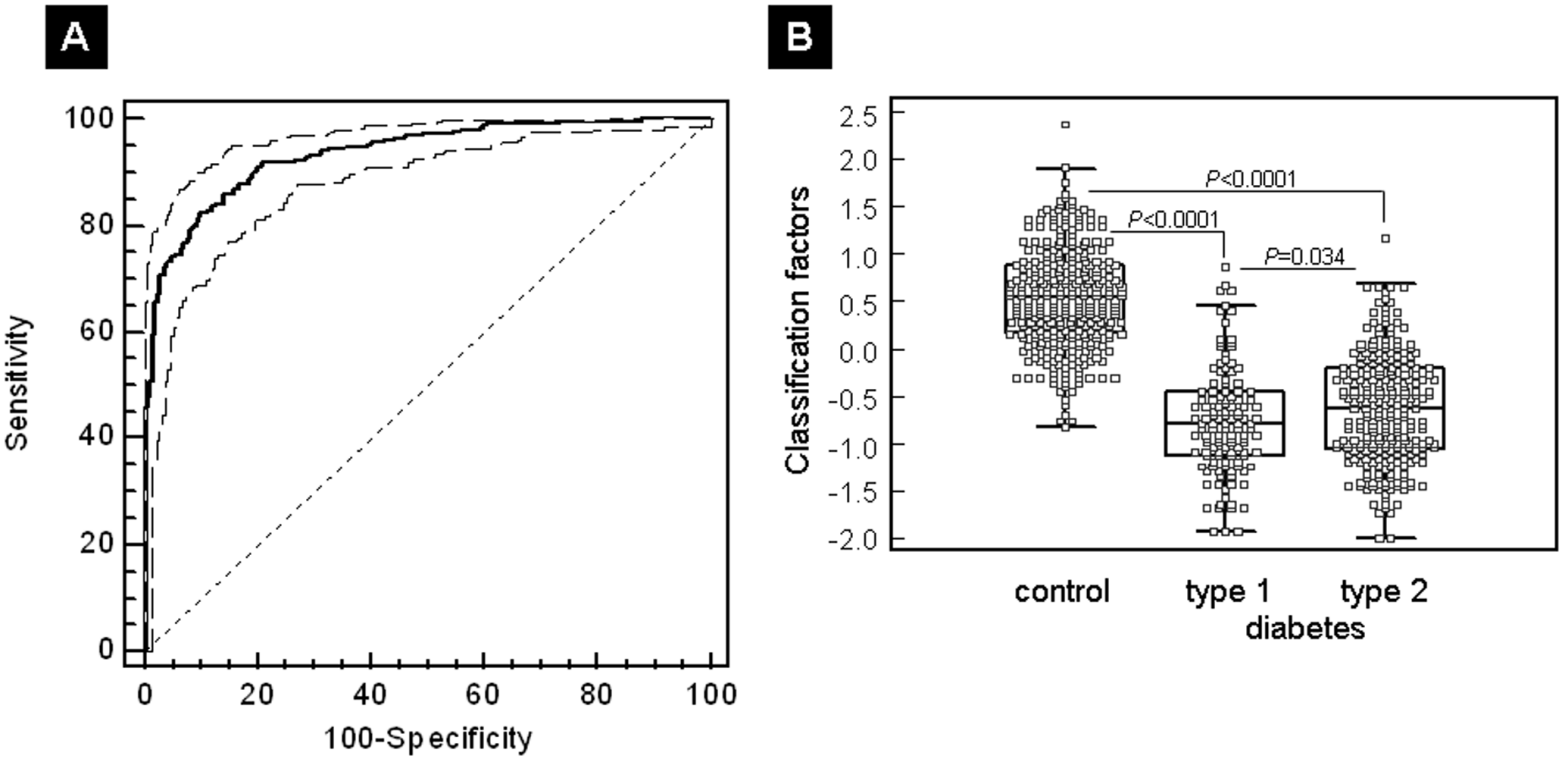
Results for validation of the urinary proteome pattern specific for diabetes. (**A**) ROC curve for the independent validation set (n = 697). ROC analysis for diagnosis of DM irrespective of diabetes type using a 261 marker panel [11]. An AUC value of 94% was calculated for the discrimination of case and control groups of the multicenter patient cohort (*P*<0.0001). (**B**) Box-and-whisker plots of SVM scores for the classified patients. Scores for each individual patient of the validation set are given as open black squares. Medians of T1D [median (interquartile range): −0.78 (−1.12 to −0.45)] and T2D [−0.63 (−1.06 to −0.21)] differed significantly (*P* = 0.034).

Recently, we identified a panel of 261 urinary biomarkers that exhibit significant differences between patients with DM and non-DM individuals [11]. In the study by Snell-Bergeon et al. [11], an SVM-derived classifier based on the DM specific panel (“diabetes 7”) was tested in a small one-center cohort of patients with T1D. In this first part of the study (A), to thoroughly validate these marker candidates in an independent multicenter validation set, we collected 697 urine samples from patients with either T1D or T2D and healthy controls in 9 additional centers. Urine samples of 382 DM and 315 non-DM were analyzed using CE-MS urinary proteome analysis, as graphically outlined in **Figure 1A**. The distribution of the 261 biomarkers in the 697 validation samples is given in **Table S1.** The established diabetes 7 model enabled classification of this independent validation cohort with an AUC in ROC analysis of 94% (95% CI: 92–95%) (**Figure 2A**). The comparison of classification scores for the non-DM control samples showed statistically highly significant differences (*P*<0.0001) compared to T1D and as well as to T2D patients (**Figure 2B** **and Table S2**). In order to further validate the individual DM biomarker candidates, we applied Mann-Whitney U-testing to identify out of the 261 peptides those which are significantly associated with DM also in the independent multicenter cohort of 697 patients. Of the 261 peptides, 148 displayed *P*≤0.05 in the validation cohort, indicating significant association with DM in this independent patient cohort.

In summary, the previously developed DM specific panel is able to identify patients with DM independent of the diabetes type. However, the AUC value of only T1D patients compared to controls is higher (0.946) than the AUC value of T2D patients (0.932). Therefore, we also compared the median scoring of T1D and T2D patients and, interestingly, it was significantly (*P* = 0.034) different.

### B. Urinary biomarkers distinguishing type 1 and type 2 DM

After successful validation of DM specific biomarkers, and initiated by the observed difference between T1D and T2D, we subsequently aimed to investigate these differences in more detail in this second part of the study (B). For this purpose we employed the urinary proteome data from the 382 DM subjects described above and additional urinary proteome data from 205 diabetic subjects previously used for DM biomarker discovery [11], a total of 587 datasets from DM subjects (299 T1D and 288 T2D, **Figure 1B**).

To avoid any interference of peptides deriving from diabetic nephropathy, we only include DM patients without any evidence for renal disease. Therefore, we used urine samples of normoalbuminuric T1D and T2D patients to identify DM type specific biomarkers. Of the 587 diabetic subjects, 369 were excluded due to evidence of chronic renal disease, and 218 had normal renal function (136 with T1D and 82 with T2D). These 218 subjects were randomly divided into a discovery set (n = 110, 68 T1D and 42 T2D) and an independent validation set (n = 108, 68 T1D and 40 T2D, see **Figure 1B** **, flow sheet, and** **Table 1**). Characteristics of patients in the discovery set (n = 110), validation set (n = 108), and the remaining patients with DM who had chronic renal impairment (n = 369) are given in **Table 2**, stratified by DM type. The statistical comparison of the single urinary peptides and proteins in the discovery data set resulted in the tentative identification of 222 potential marker candidates (see **Table S3/set I**).

**Table 1:** Patient cohort.

Clinical condition	Patients (N)	Primary Use	Secondary Use
***Discovery set***	**110**		
Diabetes type 1 with normoalbuminuria	68	Discovery set to develop diabetic type specific markers	Training set to develop DTspP
Diabetes type 2 with normoalbuminuria	42	Discovery set to develop diabetic type specific markers	Training set to develop DTspP
***Validation set***	**108**		
Diabetes type 1 with normoalbuminuria	68	Test set to evaluateDTspP	
Diabetes type 2 with normoalbuminuria	40	Test set to evaluateDTspP	
***Chronic kidney disease set***	**369**		
Diabetes type 1 with various albuminuria states	163	Validation set to evaluateDTspP	
Diabetes type 2 with various albuminuria states	206	Validation set to evaluateDTspP	
***Total Type 1 Diabetes***	**299**	125 of 299 as validation set to evaluate diabetes model	
***Total Type 2 Diabetes***	**288**	257 of 288 as validation set to evaluate diabetes model	
***Total with diabetes***	**587**		
***Healthy controls***	**315**	Validation set to evaluate diabetes model	
***Total***	**902**		

Usage of patient cohorts in this study.

**Participating centers:** (1) University of Glasgow, Glasgow, United Kingdom; (2) University of Erlangen-Nürnberg, Germany; (3) University of Melbourne, Austin Health, Heidelberg, Victoria, Australia; (4) University of Colorado Denver, Aurora, Colorado; (5) RD–Néphrologie, Montpellier, France; (6) University of Groningen, The Netherlands; (7) Steno Diabetes Center, Gentofte, Denmark; (8) Charles University, Prague, Czech Republic; (9) Harvard Medical School, Boston, Massachusetts, (10) University of Graz, Graz, Austria.

**Table 2:** Characteristics of patients with diabetes.

Variables	Discovery set			Validation set		Chronic kidney disease set	
	type 1 (n = 68)	type 2 (n = 42)	*P-*value§	type 1 (n = 68)	type 2 (n = 40)	type 1 (n = 163)	type 2 (n = 206)
Age, years	43±11**	63±9	0.0082	47±13*	62±9	46±11**	64±11
Sex [m/f]	45/23	24/18	>0.05	42/26*	28/12	85/78**	144/62
Duration of diabetes, years	27±10**	11±8	0.0008	28±12*	11±7	29±11**	15±9
Urinary albumin,µg/ml	9±10*	6±6	>0.05	9±8*	5±3	187±322**	509±790
ACR,µg albumin/mg creatinine	10±7*	7±6	>0.05	10±7*	7±4	281±414**	515±882
GFR, ml/min/1.73 m^2^	91±22*	101±27	>0.05	98±29	109±51	74±30	79±40
Systolic blood pressure, mmHg	126±17*	136±15	>0.05	129±16*	136±17	130±20**	144±18
Diastolic blood pressure, mmHg	76±9	73±11	>0.05	75±9	76±9	75±10*	78±11
BMI, kg/m^2^	27±5**	30±5	>0.05	27±6*	31±7	26±5**	32±6
Smoking status [yes/no]	12/56	8/34	>0.05	10/58	11/29	34/129	48/158
TC, mmol/l	4.6±0.9	4.8±1.4	>0.05	4.8±0.8	4.8±1.1	4.8±1.0**	4.5±1.1
HDL, mmol/l	1.5±0.5	1.4±0.5	>0.05	1.5±0.5*	1.3±0.4	1.6±0.6**	1.3±0.4
LDL, mmol/l	2.6±0.7	2.4±0.9	>0.05	2.6±0.7	2.5±0.9	2.5±0.8*	2.3±1.0
TG, mmol/l	1.3±1.4	2.1±1.4	>0.05	1.2±1.2	1.6±0.7	1.3±0.8**	2.2±1.6
Medications [yes/no]:							
HTN	25/43**	32/10	>0.05	33/35**	27/13	110/53**	191/15
Dyslipidemia	13/55**	23/19	>0.05	20/48	19/21	60/103**	153/53
Oral hypoglycemics	0/68**	27/15	>0.05	0/68**	22/18	0/163**	131/75
Insulin	68/0**	22/20	>0.05	68/0**	21/19	163/0**	125/81

Data are mean ± standard deviation. Abbreviations: m, male; f, female; ACR, albumin extraction rate; GFR, glomerular filtration rate; BMI, body mass index; TC, total cholesterol; HDL, high-density lipoprotein; LDL, low-density lipoprotein; HTN, hypertension;

*P-value < 0.05,

**P-value <0.001 for Univariate analysis;

§ logistic regression *P*-value.

The differences of biomarkers in T1D and T2D patient urine samples may be caused by different pathophysiology of the DM, but also by differences of other clinical parameters in both cohorts. For all data sets, T1D subjects were younger, had longer diabetes duration, lower systolic blood pressure and BMI, and were less likely to be treated for hypertension (HTN) or dyslipidemia than patients with T2D. All T1D patients were treated with insulin and none were treated with oral hypoglycemics, in contrast to T2D patients. We analysed whether the different variables contributed to the prediction of diabetes type. Logistic regression can be used for prediction of the probability of occurrence of an event and makes use of several predictor variables that may be either continuous or categorical. Therefore, logistic regression was utilized to assess if demographic or clinical data, or medication use differed by DM type. For this analysis the discovery set was used. The results revealed that the prediction of DM type was not significantly dependent on gender, urinary albumin, ACR, GFR, systolic and diastolic blood pressure, BMI, smoking status, TC, HDL, LDL, TG and medication status. Of all included parameters, only age and duration of DM were significantly independently associated with DM type.

To correct the 222 marker candidates for age and duration of DM related proteomic changes, we performed a non-parametric analysis of the variances (Kruskall-Wallis test). The analysis identified 91 peptides significantly correlated with age (see **Table S3/set I**), and one peptide correlated with duration of DM, which was also among the 91 peptides correlated with age. These 91 peptides were excluded from the list of potentially diabetes type-associated biomarkers.

**Figure 3:**
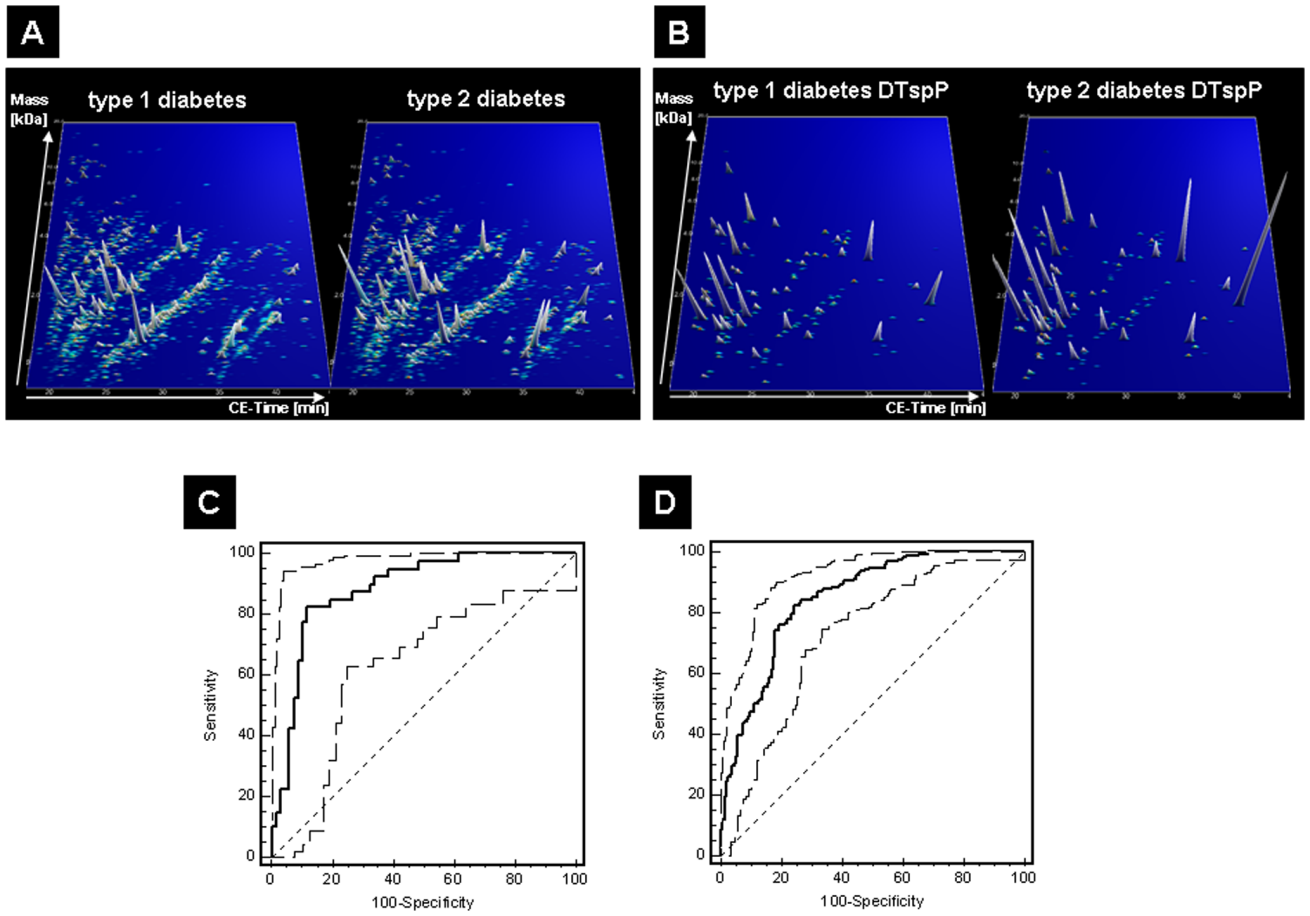
Development of diabetes type specific urinary biomarker pattern. (**A**) Compiled urinary protein profiles of patients with T1D (n = 68) and T2D (n = 42) included in the discovery set. Normalized molecular weight (800–20,000 Da) in logarithmic scale is plotted against normalized migration time (18–45 min). The mean signal intensity of polypeptides is given as peak height. (**B**) 3-D contour plots of the 131 DM type specific markers in the T1D and T2D patient cohort with 3x zoom compared to (A). ROC curves for differentiation of T1D and T2D in an independent validation set of T1D and T2D patients without clinical evidence of renal dysfunction (n = 108, AUC: 88%, **C**) and patients with evidence for renal dysfunction (n = 369, AUC: 85%, **D**).

The remaining 131 age and DM duration independent candidate biomarkers (**Table S3/set II,** **Figure 3B**) were employed in SVM-based classifier, which was trained as potentially ‘diabetes type specific polypeptide panel’ (DTspP) in the discovery set.

Subsequently, DTspP was evaluated in the validation set (n = 108) consisting of 68 normoalbuminuric T1D and 40 T2D patients samples. As shown in **Figure 3C**, the corresponding ROC analysis resulted in an AUC value of 88% (95%-CI of 81–94%). Urine samples of both cohorts (discovery and validation set) were derived from patients without any measurable renal function loss. To verify if DN could interfere in the discrimination between T1D and T2D patients, the DTspP was applied to a further cohort of DM subjects with impaired renal function (n = 369). This classification resulted in an AUC in ROC analysis (**Figure 3D**) of 85% (95% CI of 81–88%; classification factors are listed in **Table S2**).

To identify those peptides significantly differentiating T1D and T2D patients in the validation cohort without (n = 108) and with (n = 369) renal impairment, we applied Mann-Whitney U-testing in these cohorts. This held true in the validation set for 70 markers in the normoalbuminuric patients group and 86 peptides in the kidney disease cohort. 57 candidates were significant in both groups (*P*<0.05) (**Table S3/set II**).

### Sequencing of DM specific and DM type specific biomarkers

**Figure 4:**
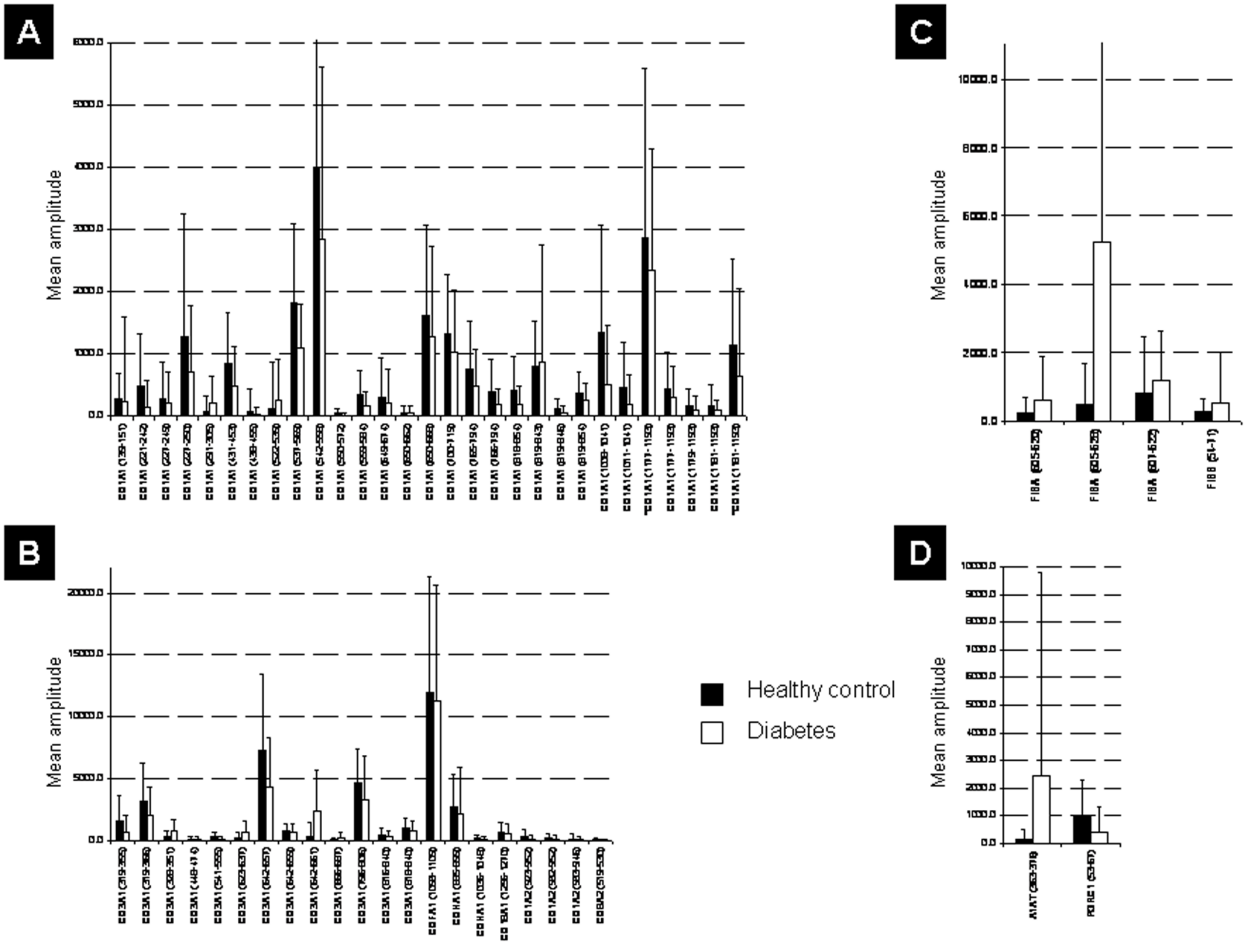
Regulation of diabetes peptide markers statistically significant in the multicenter validation set. Given are SwissProt accession names. (**A**) Regulation of collagen alpha 1 type I fragments. For two collagen fragments hydroxylated forms were identified (marked with asterisk *). (**B**) Regulation of others collagen fragments. (**C**) Regulation of fibrinogen alpha fragments. (**D**) Regulation of other identified peptide fragments.

**Figure 5:**
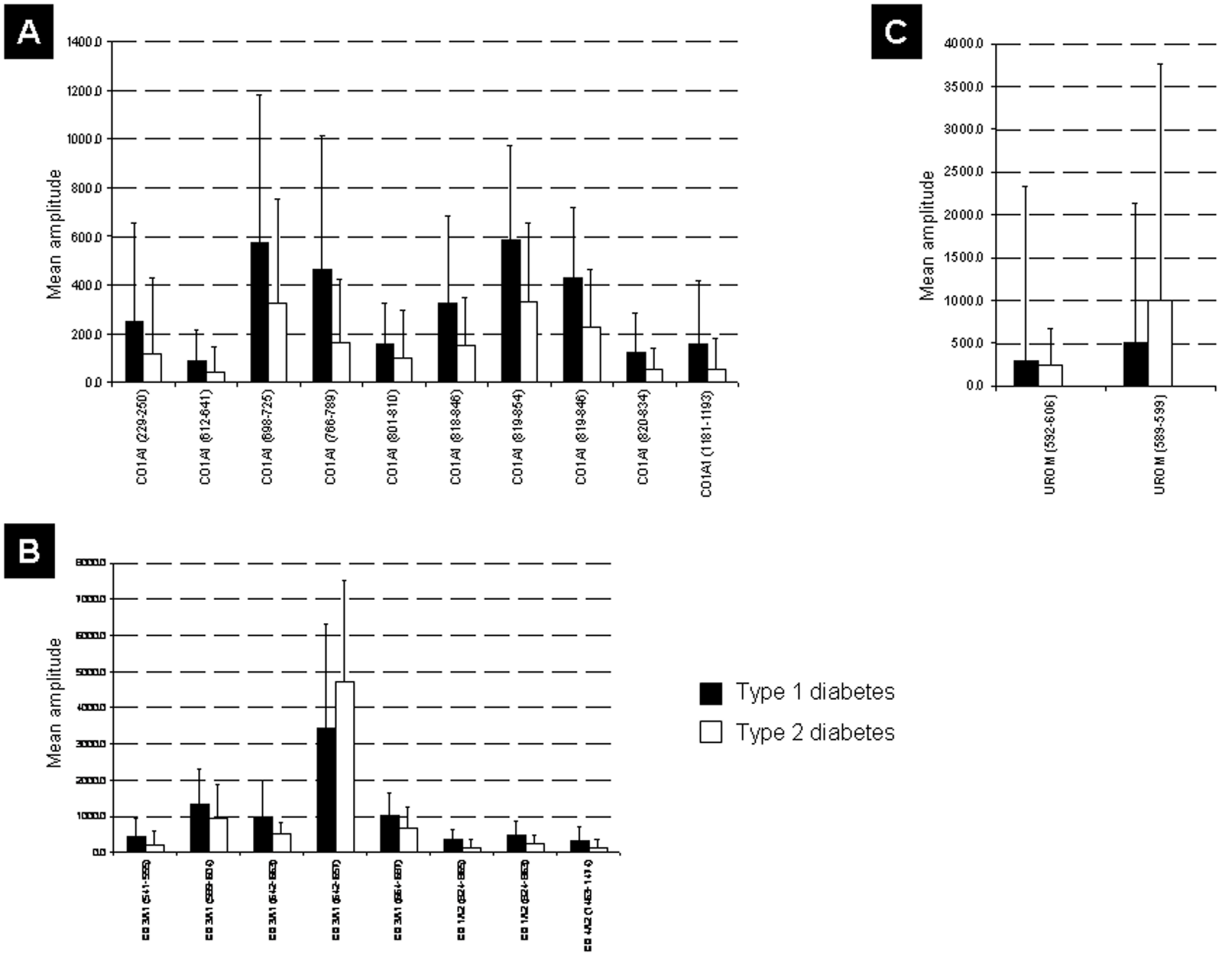
Regulation of identified statistically significant peptide markers for diabetes type in the discovery set. Given are SwissProt accession names. (**A**) Regulation of collagen alpha 1 (I) fragments. (**B**) Regulation of other types of collagen fragments. (**C**) Regulation of uromodulin fragments.

We applied tandem mass spectrometry to obtain peptide sequences. We successfully obtained sequences for 100 of the 261 DM biomarkers and 40 of 131 DM type specific peptides (**Table S1 and S3/set II**). Of the validated 148 DM markers and the 57 DM type specific biomarkers we were able to identify 56 and 20 peptides, respectively. **Table S4** displays sequence and information on the regulation of the identified and validated biomarkers for DM. The regulation of these markers in urine of DM patients and healthy controls is also shown in **Figure 4**. The validated and sequenced DM type-specific markers are listed in **Table 3** **,** and their regulation between T1D and T2D patients are shown in **Figure 5**.

**Table 3:** Identified and validated diabetes type markers.

Protein ID	Mass (Da)	CE-time (min)	*P*-values			F	Sequence	Protein name	Accession number
			Discovery set	Validation set	CKD set				
12998	1009.45	27.27	2.04E-02	5.30E-04	3.84E-03	−1.6	DRGEpGPpGP	Collagen alpha-1 (I) chain	gi124056487
20072	1134.58	23.66	5.02E-03	1.74E-02	3.34E-06	−2.3	PIGQEGAPGRPG	Collagen alpha-2 (IV) chain	gi143811377
20756	1141.54	37.33	4.41E-02	1.31E-03	8.34E-06	−2.8	GPpGpPGPPGPPS	Collagen alpha-1 (I) chain	gi124056487
21919	1159.6	26.07	1.69E-02	4.27E-02	2.08E-08	2.0	SGSVIDQSRVL	Uromodulin	gi137116
32471	1326.56	27.11	2.32E-02	5.19E-03	7.84E-09	−2.1	SpGGpGSDGKpGPpG	Collagen alpha-1 (III) chain	gi124056490
34154	1357.58	30.02	4.49E-02	2.94E-03	4.34E-09	−2.4	DGQpGAKGEpGDAGA	Collagen alpha-1 (I) chain	gi124056487
38798	1438.67	27.88	2.96E-02	3.78E-02	1.01E-02	1.4	GLpGTGGPpGENGKpG	Collagen alpha-1 (III) chain	gi124056490
40260	1451.69	22.55	2.76E-02	5.47E-03	4.15E-03	−1.4	ApGKNGERGGpGGpGP	Collagen alpha-1 (III) chain	gi124056490
54448	1679.95	24.79	2.97E-04	4.08E-04	2.11E-04	−1.2	VIDQSRVLNLGPITR	Uromodulin	gi137116
62504	1846.85	32.06	2.87E-03	2.59E-04	7.30E-07	−2.8	TGPIGPpGPAGApGDKGESGP	Collagen alpha-1 (I) chain	gi124056487
70911	2019.95	24.62	5.57E-03	1.86E-05	5.06E-09	−1.8	GLpGTGGPpGENGKpGEPGpKG	Collagen alpha-1 (III) chain	gi124056490
73177	2062.93	26.58	8.68E-03	9.34E-03	1.15E-02	−1.5	DAGApGAPGGKGDAGApGERGPpG	Collagen alpha-1 (III) chain	gi124056490
80012	2191.99	22.39	2.11E-02	5.95E-04	2.81E-03	−2.2	NGDDGEAGkPGRpGERGPpGPQ	Collagen alpha-1 (I) chain	gi124056487
88282	2339	34.01	2.20E-02	1.09E-02	1.14E-04	−1.8	GANGApGNDGAKGDAGApGApGSQGApG	Collagen alpha-1 (I) chain	gi124056487
92841	2430.1	28.33	3.21E-03	2.00E-04	1.47E-09	−1.9	ADGQPGAKGEpGDAGAKGDAGPpGPAGP	Collagen alpha-1 (I) chain	gi124056487
94948	2487.13	28.27	3.34E-02	1.61E-02	2.17E-04	−2.2	GADGQPGAKGEpGDAGAKGDAGPpGPAGP	Collagen alpha-1 (I) chain	gi124056487
105105	2687.22	28.99	2.87E-02	6.52E-04	3.90E-03	−2.2	KDGEAGAQGPpGPAGPAGERGEQGPAGSpG	Collagen alpha-1 (I) chain	gi124056487
121775	3092.46	31.25	2.96E-03	3.11E-03	3.32E-10	−1.8	ADGQPGAkGEPGDAGAKGDAGPPGPAGpAGpPGPIG	Collagen alpha-1 (I) chain	gi124056487
139064	3616.72	33.19	2.63E-03	1.67E-05	2.65E-05	−2.1	DQGPVGRTGEVGAVGPPGFAGEkGPSGEAGTAGPpGTpGP	Collagen alpha-2 (I) chain	gi124056488
143947	3801.77	33.46	2.38E-03	2.73E-04	3.29E-07	−2.3	DQGPVGRTGEVGAVGPpGFAGEKGPSGEAGTAGPpGTpGPQG	Collagen alpha-2 (I) chain	gi124056488

20 identified and validated diabetes type marker. Shown are the protein/peptide identification number in the dataset (Protein ID), mass (in Da) and normalized igration time (in min), the adjusted P-values using Benjamini-Hochberg (BH) for training data and unadjusted *P*-values using Mann-Withney U-test for validation and CKD cohorts, regulation factor (F) for type 2 diabetes compared to type 1 diabetes [for mean(D2T)>mean(D1T): mean(D2T)/mean(D1T); for mean(D2T)<mean(D1T): -mean(D1T)/mean(D2T)]. In addition, sequences (modified amino acids: p = hydroxyproline; k =  hydroxylysine; m = oxidized methionine), protein names and accession numbers are given.

The majority of the identified biomarkers were fragments of collagen alpha-1 (I) and (III). In general, collagen fragment levels were decreased in urine of patients with DM compared to non-DM subjects (**Figure 4A and B**), with even further decreased levels in urine of T2D compared to T1D patients (**Figure 5A and B**). Most of these collagen fragments are C-terminal. In contrast, fragments of fibrinogen alpha and beta were increased in the urine of patients with DM compared to non-DM subjects (**Figure 4C**). Furthermore, fragments of alpha-1-antitrypsin, membrane-associated progesterone receptor component 1 and uromodulin (for regulation see **Table S4, 4**, **Table S1 or S3/set II** and Figure **4D** **,** **5C**) were among the biomarkers.

## Discussion

This study represent the largest proteomic study (with respect to cohort size) reported to date. Furthermore, this is the first study to our knowledge which is dealing with the investigation of differences between the urinary proteome of T1D and T2D patients. In this work we successfully validated urinary peptides that are specific for DM in general (part A), and further identified urinary peptides significantly associated with T1D or T2D (part B). The defined biomarkers indicate (patho)physiological differences in the extracellular remodeling of T1D and T2D. Due to different etiopathologies of T1D and T2D, T1D subjects in our study were significantly younger, and had significantly longer duration of DM. In addition, all T1D subjects received insulin treatment. All these potential confounding factors were considered in the statistical analysis, and peptides which were significantly associated with these factors were excluded from further examinations.

The most prominent DM associated urinary proteome changes were a significant reduction of specific collagen alpha-1 (I) and (III) fragments, and in direct comparison among patients without evidence of chronic kidney disease, these changes were significantly more pronounced in T2D than in T1D, despite the lower ACR and higher estimated GFR in T2D patients. This corresponds to the morphological observation that along with increased β-cell apoptosis, pancreatic islets from T2D patients contain amyloid deposits and resulting fibrosis [12], [13]. In this context it is worth mentioning that extracellular matrix (ECM) homeostasis is maintained by the balance between tissue inhibitor of metalloproteinases (TIMP) and matrix metalloproteases (MMP). Decreased activity of certain MMPs (e.g. MMP-2, -3, and -9), as described in diabetes [14]–[16], would account for our finding of decreased urinary excretion of collagen fragments, since less collagen filaments would in this case be cleaved from the ECM. Our data support the hypothesis that physiological degradation of ECM components, especially collagen fibers, may be disturbed as a result of DM and this phenomenon would subsequently result in morphologically observed increased ECM deposits [15]–[17]. These data indicate a demand for further research to investigate the detailed relationship between MMPs/TIMPs/ECM in DM-associated complications in a systems approach, as recently suggested [18], [19].

In addition, the data on the differences in urinary collagen fragments may indicate that the mechanism of attenuation of collagen degradation is different in T1D and T2D. In addition to MMPs, advanced glycemic end products (AGEs) are prominent candidates possibly responsible for a disturbance in collagen breakdown and chemical modification of collagen [20], [21]. While we could not find reports indicating significant differences in protease activity or AGE status between T1D and T2D, both phenomena have been observed when comparing patients with diabetes to normal controls [9], [22]. Based on the data reported here, we hypothesize that the underlying molecular changes that result in vascular damage and fibrosis in diabetes may be different between T1D and T2D, as indicated by significant differences in urinary collagen fragments.

Alpha-1-antitrypsin (AAT) is a member of the serpin family, a major acute phase protein, and a physiological inhibitor of serin proteases like neutrophil elastase, resulting in a plethora of various anti-inflammatory and anti-apoptotic effects [23]. Plasma levels and activity of AAT are reported to be significantly decreased in DM patients [24]–[26], while we and others found urinary fragments of AAT to be significantly increased [27], suggesting increased degradation and subsequent renal clearance of AAT-derived peptides in DM. Increased degradation, resulting in decreased AAT serum levels, would facilitate conversion of fibrinogen to fibrin by thrombin and release of fibrinogen-alpha and –beta. This assumption is supported by the observed increase of urinary fibrinogen-alpha and –beta-chain fragments in diabetics compared to controls, and consistent with the the significant pro-thrombotic risk in DM observed by others [28].

Progesterone receptor membrane component 1 (PGRMC1) is a member of the so-called membrane-associated progesterone receptors (MAPR) [29]. As an adaptor protein, PGRMC1 was proposed to be involved in regulating protein interactions, intracellular signal transduction and/or membrane trafficking [29]. Interestingly, in the rat, PGRMC1 activation by progesterone is discussed as an inhibitor of cell respiration and suppressor of glucose transport in late rodent pregnancy [30]. This effect could contribute to pregnancy associated changes in glucose homeostasis in gestational diabetes.

While uromodulin has previously been reported to be decreased in patients with DM [31]–[33], we observe the up-regulation of a uromodulin fragment without a C-terminal arginine residue (**Table 3**). This may be a result of increased proteolytic activity in DM, resulting in decrease of the parental protein, but increase in degradation products. However, this hypothesis requires further investigation.

Several approaches aiming at the analysis of differently regulated proteins in body fluids from patients with T1D and T2D have been performed [31], [34]. One early proteomic approach using fractionated human serum samples in the context of T2D and insulin resistance was performed by Zhang et al. [34] to mine low abundant proteins. When comparing serum from patients with T2D or insulin resistance to controls' serum, haptoglobin was elevated. Also, several other proteins involved in the inflammatory response, like α-2 macroglobulin, fibrinogen, complement C3 and C1 inhibitor were altered. Many of the detected proteins have been connected to DM, such as the acute phase protein haptoglobin, which has been associated with cardiovascular and renal complications in T1D [35], [36]. However, we are not aware of any investigation using urine for the analysis of differences in the proteome of T1D versus T2D.

Our study has some potential limitations. Health-care provider definitions of diabetes type were used, and although standard clinical methodology was used by experienced diabetologists, tests such as T1D-specific auto-antibodies were not performed. However, any possible misclassification of subjects by diabetes type would bias our findings toward the null. Additionally, as expected, the T1D subjects had different clinical and demographic characteristics compared to the T2D subjects. Therefore, we adjusted for these differences in the statistical analyses to avoid introduction of bias. Although we used state-of-the-art tandem mass spectrometry to identify discovered biomarker candidates by peptide sequence, we were unable to sequence all biomarker candidates. Most likely, we have reached the technical limits of currently available sequencing technology of naturally occurring peptides [37]. In general, native peptide sequencing is limited by post-translational modifications, complicating not only peptide ion fragmentation, but also subsequent database searches [37], [38]. Additionally, the proteomics CE-MS technology is able to detect polypeptides with a high analytical sensitivity [39], [40], whereas tandem mass spectrometry used for sequencing has higher detection limits [41], [42].

In conclusion, this work gives clear and valid evidence, based on a multicenter cohort, of differences in the urinary proteome of T1D versus T2D patients with normal renal function, validated also in those with chronic kidney disease. Future studies should enable identification of not yet sequenced differentially expressed peptides and determine how these differences can be exploited for disease monitoring and therapeutic issues. However, the vast amount of data reported here and available today clearly suggest that alterations in the remodeling of extracellular matrix, and likely in endogenous proteolytic activity, are among the hallmarks of DM. These pathophysiological changes likely represent promising targets for pharmacological intervention, aiming specifically at prevention of diabetes-associated vascular complications. Further, the alterations in urinary ECM degradation products show significant differences between T1D and T2D.

## Materials and Methods

### Patient characteristics and study design

Urine samples were collected as described previously [43], in agreement with the protocol established by HUPO (www.hupo.org/research/urine) and EuroKUP (www.eurokup.org). In short, urine samples were collected using standard operation procedures and frozen immediately without the addition of any preservatives. 587 patients with either T1D (n = 299) or T2D (n = 288) were recruited at 10 different hospital centers in the US, Europe, and Australia (see **Table 1** for details). The diagnosis of T1D and T2D was based on commonly accepted diagnostic criteria [44]. The pre-existing diagnosis of T1D and T2D as assigned in each center was considered as reference-standard for the purpose of comparison with the generated DM-specific urinary polypeptide panels. 205 of the 587 diabetes patients were previously used [11] for development of DM-specific panel. These remaining 382 DM samples and additional 315 samples from healthy non-DM controls were used in this study as an initial step to validate the DM (yes/no) panel (53% male, mean age±SD, 40±10 years) [45], [46] (**Figure 1**).

Chronic renal impairment was assessed using albumin/creatinine ratio >30 mg/g, or with a glomerular filtration rate (GFR) <60 unit, and scoring negative in a previously published classification model for chronic kidney disease [47]. The Cockcroft-Gault method was used to estimate GFR.

### Sample preparation

A 0.7 mL aliquot of urine was thawed immediately before use and diluted with 0.7 mL 2 M urea, 10 mM NH_4_OH containing 0.02% SDS. In order to remove high molecular weight compounds of urine, samples were filtered using Centrisart ultracentrifugation filter devices (20 kDa molecular weight cut-off; Sartorius, Goettingen, Germany) at 3,000 g until 1.1 mL of filtrate was obtained. Subsequently, filtrate was desalted using a PD-10 column (GE Healthcare, Sweden) equilibrated in 0.01% NH_4_OH in HPLC-grade water. Finally, samples were lyophilized and stored at 4°C. Shortly before CE-MS analysis, lyophilisates were resuspended in HPLC-grade water to a final protein concentration of 0.8 µg/µL as verified by BCA assay (Interchim, Montlucon, France).

### Urinary proteome analysis

CE-MS analysis was performed as described previously [2], [40]. By this procedure the average recovery rate in the preparation procedure was ∼85% and the limit of detection was ∼1 fmol [40]. Mass resolution was controlled to be above 8,000 enabling resolution of monoisotopic mass signals for z≤6. After charge deconvolution, mass deviation was <25 ppm for monoisotopic resolution and <100 ppm for unresolved peaks (z>6). The analytical precision of the set-up was assessed by reproducibility achieved for repeated measurements of the same replicate and by the reproducibility achieved for repeated preparations and measurements of the same urine sample [40]. To ensure high data consistency, a minimum of 950 peptides/proteins had to be detected with a minimal MS resolution of 8,000 in a minimal migration time interval of 10 minutes. By following this set-up, CE-MS enabled reproducible and robust high-resolution urinary proteome analysis.

### Data processing

Mass spectral ion peaks representing identical molecules at different charge states were deconvoluted into single masses using MosaiquesVisu software [48]. For noise filtering, signals with z>1 observed in a minimum of 3 consecutive spectra with a signal-to-noise ratio of at least 4 were considered. MosaiquesVisu employs a probabilistic clustering algorithm and uses both isotopic distribution (for z≤6) as well as conjugated masses for charge-state determination of peptides/proteins. The resulting peak list characterizes each polypeptide by its mass and its migration time. TOF-MS data were calibrated utilizing 80 reference masses exactly determined by Fourier transform ion cyclotron resonance mass spectrometry (FT-ICR-MS). For calibration, linear regression is performed. Both capillary electrophoresis (CE)-migration time and ion signal intensity (amplitude) show variability, mostly due to different amounts of salt and peptides in the sample and were consequently normalized. Reference signals of more then 1700 urinary polypeptides were used for CE-time calibration by local regression [49]. For normalization of analytical and urine dilution variances, MS signal intensities were normalized relative to 29 “housekeeping” peptides with small relative standard deviation. For calibration, local regression is performed [50]. The obtained peak lists characterized each polypeptide by its molecular mass [Da], normalized CE migration time [min] and normalized signal intensity. To avoid artifacts (specific individual peptides) only detected peptides with frequency >20% were deposited, matched, and annotated in a Microsoft SQL database allowing further statistical analysis. For clustering, peptides in different samples were considered identical, if mass deviation was ≤50 ppm for small or ≤75 ppm for larger peptides. Due to analyte diffusion effects, CE peak widths increase with CE migration time. In the data clustering process this effect was considered by linearly increasing cluster widths over the entire electropherogram (19 min to 45 min) from 2–5%. After calibration, mean deviation of migration time was controlled to be below 0.35 minutes. All annotated data are available in **Table S5C**.

### Statistical analysis

#### Patients' data analysis

Logistic regression (MedCalc version 8.1.1.0, MedCalc Software, Belgium, www.medcalc.be) was used to assess if diabetes type might be predicted from clinical data (**Table 2**).

#### Biomarker discovery

Peptides' *P*-values were calculated using the base 10 logarithm transformed intensities and the Gaussian approximation to the t-distribution. For multiple testing corrections, *P*-values were corrected using the false discovery rate (FDR) procedure introduced by Benjamini and Hochberg [51]. The FDR is the fraction of false positives among all tests declared significant. FDR was controlled to be ≤0.05, which means that on average less than 5% of peptides declared significant are actually false positives. On the other hand, the other 95% of the biomarkers were indeed true positives. The approach is reported to have high statistical power for biomarker discovery in the situation of differential expression between two samples, when subjected to two different treatments, such as disease/no disease [51]. Only proteins that were detected in a diagnostic group of patients in at least 50% of samples were considered for testing. The test was implemented as macros in SAS (www.sas.com) and is part of the multitest R-package (www.bioconductor.org). Non-parametric Kruskal-Wallis one-way analysis of variances [52] (MedCalc version 8.1.1.0, MedCalc Software, Belgium, www.medcalc.be) was used to assess of candidates' dependency on age- and DM duration.

#### Descriptive statistics

Sensitivity, specificity, and 95% confidence intervals (95% CI) were calculated using receiver operating characteristic (ROC) plots [53] (MedCalc version 8.1.1.0, MedCalc Software, Belgium, www.medcalc.be). The receiver operating characteristic curve (ROC) was obtained by plotting all sensitivity values (true positive fraction) on the y-axis against their equivalent (1-specificity) values (false positive fraction) for all available thresholds on the x-axis. The area under the ROC curve (AUC) provides the single best measure of overall accuracy independent of any threshold.

### Classification

Disease specific protein/peptide patterns were generated using support vector machine-based (SVM) MosaCluster software [4]. SVM view a data point (proband's urine sample) as a p-dimensional vector (p numbers of protein used in the pattern), and they attempt to separate them with a (p − 1) dimensional hyperplane. The hyperplane with the maximal distance from the hyperplane to the nearest data point is selected. Classification is performed by determining the Euclidian distance (defined as the SVM score) of the polypeptides to the (n-1) dimensional maximal margin hyperplane and the direction of the vector.

### Sequencing of peptides

In order to identify the defined biomarkers, we applied MS/MS peptide sequencing using CE- or liquid chromatography (LC)-MS/MS analysis including either collision-induced dissociation (CID) [8], [54] or electron transfer dissociation (ETD) [38], [55], [56]. Obtained MS/MS data were submitted to MASCOT (www.matrixscience.com) for search against human entries in the MDSB Protein Database. Accepted parent ion mass deviation was 0.5 Da; accepted fragment ion mass deviation was 0.7 Da. Hits were accepted with MASCOT peptide scores of ≥20. Additionally, ion coverage was controlled to be related to main spectral fragment features (b/y or c/z ion series). If necessary, manual de novo sequencing was performed to confirm the identifications. The number of basic and neutral polar amino acids of the peptide sequences was utilized to correlate peptide sequencing data to CE-MS data, as described [54].

## Supporting Information

Table S1 261 DM-specific peptides included in diabetes7 model. Shown are the protein/peptide identification number in the dataset (Protein ID), mass (in Da) and normalized migration time (in min), the p-values [unadjusted using Mann-Withney U-test], frequency, mean amplitude and standard deviation in the two groups of the cohort, and the regulation factor by diabetes compared to healthy controls. In addition, sequences (modified amino acids: p = hydroxyproline; k =  hydroxylysine; m = oxidized methionine), protein names, start and stop amino acid, Swiss-Prot entries and accession numbers are given.
(0.11 MB XLS)

Table S2 Classification results by application of the models for diabetes (diabetes 7) and diabetes type (DTspP). In this table patient IDs of all included patients are listed in combination with diagnosis, usage in this study, and classification results. For training set of DTspP total cross validated data are given.
(0.11 MB XLS)

Table S3 “Set II”: 131 peptides included in DTspP. Shown are the protein/peptide identification number in the dataset (Protein ID), mass (in Da) and normalized migration time (in min), the adjusted P-values using Benjamini-Hochberg (BH) for training data and unadjusted P-values using Mann-Withney U-test for validation and CKD cohorts, the frequency, mean amplitude, standard deviation in the two groups of diabetes in the training set and in the group of 315 healthy controls, and the regulation factor for type 1 compared to type 2 and type 1 and 2 diabetes compared to healthy controls. In addition, sequences (modified amino acids: p = hydroxyproline; k =  hydroxylysine; m = oxidized methionine), protein names, start and stop aino acid, Swiss-Prot entries and accession numbers are given.
(0.10 MB XLS)

Table S4 Identified and validated diabetes markers.
(0.12 MB DOC)

Table S5 Pivot Table includes all CE-MS data for all samples in the study. Given are the mass (in Da) and migration time (in min) of peptides assigned to a certain Protein ID, which is subsequently utilized as unique identifier in the database. Sample assignment indicates the unique patient ID. The table lists the amplitudes of each polypeptide in the individual samples.
(3.69 MB ZIP)
