# Supplementary material for: Urinary Collagen Fragments Are Significantly Altered in Diabetes: A Link to Pathophysiology
Source: PLoS One. 2010 Sep 28;5(9):e13051. doi: 10.1371/journal.pone.0013051 (PMC2946909; doi:10.1371/journal.pone.0013051)
Supplement: Table S4 — Identified and validated diabetes markers. (0.12 MB DOC) [file pone.0013051.s004.doc]

**SupplementaryTable S4:** Identified and validated diabetes markers.

| **Protein ID*** | **Mass (Da)** | **CE-time (min)** | **U-Test *P-*value** | **F** | **Sequence** |  | **Protein name** | **Accession number** |
| --- | --- | --- | --- | --- | --- | --- | --- | --- |
| 15954 | 1070.49 | 36.49 | 4.87E-04 | -1.6 | GPpGpPGPpGPP |  | Collagen alpha-2 (VIII) chain | gi45644957 |
| 18943 | 1114.49 | 25.55 | 2.47E-13 | -1.4 | SpGERGETGPp |  | Collagen alpha-1 (III) chain | gi124056490 |
| 20756 | 1141.54 | 37.33 | 1.43E-03 | -1.7 | GPpGpPGPPGPPS |  | Collagen alpha-1 (I) chain | gi124056487 |
| 20862 | 1143.52 | 36.97 | 2.19E-08 | -1.2 | GLPGPpGPpGPpG |  | Collagen alpha-1 (I) chain | gi124056487 |
| 21747 | 1157.54 | 37.44 | 3.20E-10 | -1.8 | GPPGPpGppGPPS |  | Collagen alpha-1 (I) chain | gi124056487 |
| 22835 | 1173.53 | 37.49 | 2.37E-08 | -2.1 | GPpGPpGPpGPVT |  | Collagen alpha-1 (XVII) chain | gi146345399 |
| 25053 | 1211.54 | 25.82 | 2.02E-02 | -1.2 | GPpGEAGKpGEQG |  | Collagen alpha-1 (I) chain | gi124056487 |
| 32343 | 1324.59 | 28.70 | 2.42E-17 | 3.0 | TGPGGDKGDTGPpGP |  | Collagen alpha-1 (III) chain | gi124056490 |
| 32470 | 1326.55 | 29.20 | 4.44E-16 | -2.7 | SpGGpGSDGKpGPpG |  | Collagen alpha-1 (III) chain | gi124056490 |
| 33776 | 1351.64 | 38.76 | 7.58E-03 | -1.5 | PpGPpGPpGPPGPPS |  | Collagen alpha-1 (I) chain | gi124056487 |
| 34766 | 1367.64 | 38.88 | 7.20E-06 | -1.3 | PpGPpGPpGPPGTPV |  | Collagen alpha-1 (XVIII) chain | gi215274264 |
| 36769 | 1405.64 | 20.14 | 8.00E-06 | -3.2 | DGPpGRDGQpGHKG |  | Collagen alpha-2 (I) chain | gi124056488 |
| 37903 | 1424.66 | 39.30 | 1.53E-07 | -1.2 | GLPGPpGPpGSFLSN |  | Collagen alpha-1 (XVII) chain | gi55957394 |
| 38798 | 1438.67 | 27.88 | 5.29E-13 | -1.7 | GLpGTGGPpGENGKpG |  | Collagen alpha-1 (III) chain | gi124056490 |
| 38879 | 1439.66 | 29.82 | 1.03E-25 | 17.0 | TIDEKGTEAAGAMF |  | Alpha-1-antitrypsin | gi1703025 |
| 40541 | 1458.63 | 27.94 | 2.35E-03 | 2.4 | SpGENGApGQmGPRG |  | Collagen alpha-1 (I) chain | gi124056487 |
| 42594 | 1491.74 | 39.83 | 3.13E-02 | -1.5 | VGPpGpPGPPGPPGPPS |  | Collagen alpha-1 (I) chain | gi124056487 |
| 43442 | 1507.74 | 40.02 | 2.70E-02 | -1.2 | VGPpGPpGPpGPPGPPS |  | Collagen alpha-1 (I) chain | gi124056487 |
| 43543 | 1508.68 | 29.33 | 2.85E-10 | -1.4 | GSpGSpGPDGKTGPPGp |  | Collagen alpha-1 (I) chain | gi124056487 |
| 44618 | 1523.74 | 40.66 | 1.05E-02 | -1.1 | GDPGPPGpPGpPGpPAI |  | Collagen alpha-1 (XV) chain | gi68839886 |
| 49958 | 1608.73 | 30.93 | 2.77E-12 | -2.4 | SGDSDDDEPPPLPRL |  | Membrane-associated progesterone receptor component 1 | gi6647589 |
| 50172 | 1612.76 | 23.38 | 7.10E-08 | -3.1 | ApGSKGDTGAKGEpGPVG |  | Collagen alpha-1 (I) chain | gi124056487 |
| 52100 | 1638.73 | 20.23 | 2.18E-08 | 1.4 | AGSEADHEGTHSTKRG |  | Fibrinogen alpha chain | gi1706799 |
| 53216 | 1654.78 | 23.13 | 2.41E-08 | 2.2 | SpGEAGRpGEAGLpGAKG |  | Collagen alpha-1 (I) chain | gi124056487 |
| 53633 | 1664.75 | 29.81 | 3.54E-02 | -1.1 | GLpGTGGPpGENGKPGEp |  | Collagen alpha-1 (III) chain | gi124056490 |
| 53957 | 1669.69 | 21.46 | 1.45E-05 | 2.4 | DEAGSEADHEGTHSTK |  | Fibrinogen alpha chain | gi1706799 |
| 55582 | 1697.74 | 30.88 | 9.11E-08 | -1.3 | NGAPGNDGAKGDAGAPGAPG |  | Collagen alpha-1 (I) chain | gi124056487 |
| 58941 | 1765.81 | 31.00 | 4.77E-04 | -1.3 | GPpGEAGKpGEQGVpGDLG |  | Collagen alpha-1 (I) chain | gi124056487 |
| 60126 | 1793.88 | 32.37 | 2.31E-10 | 2.1 | EEAPSLRPAPPPISGGGY |  | Fibrinogen beta chain | gi56749856 |
| 61304 | 1818.83 | 30.95 | 3.59E-13 | 6.6 | GLpGTGGPpGENGKPGEPGp |  | Collagen alpha-1 (III) chain | gi124056490 |
| 64887 | 1892.86 | 24.33 | 1.04E-09 | 4.2 | GApGApGGKGDAGApGERGPpG |  | Collagen alpha-1 (III) chain | gi124056490 |
| 73697 | 2070.92 | 25.40 | 5.72E-13 | -1.7 | GNSGEpGApGSKGDTGAKGEPGp |  | Collagen alpha-1 (I) chain | gi124056487 |
| 73913 | 2076.95 | 21.78 | 1.09E-14 | -3.1 | GPpGPpGKNGDDGEAGkpGRPG |  | Collagen alpha-1 (I) chain | gi124056487 |
| 76839 | 2128.98 | 26.97 | 9.28E-12 | -4.3 | DGKTGpPGPAGQDGRPGPpGppG |  | Collagen alpha-1 (I) chain | gi124056487 |
| 77184 | 2137.94 | 21.79 | 6.12E-07 | -1.3 | NGEpGGKGERGApGEKGEGGpPG |  | Collagen alpha-1 (III) chain | gi124056490 |
| 78792 | 2168.97 | 32.91 | 7.12E-16 | 2.1 | SDGQpGPpGPpGTAGFpGSpGAKG |  | Collagen alpha-1 (III) chain | gi124056490 |
| 79786 | 2189.00 | 26.89 | 2.61E-05 | 1.1 | ADGQPGAKGEPGDAGAKGDAGPpGP |  | Collagen alpha-1 (I) chain | gi124056487 |
| 80306 | 2194.97 | 20.17 | 2.00E-15 | -2.5 | NDGPpGRDGQpGHKGERGYpG |  | Collagen alpha-2 (I) chain | gi124056488 |
| 83577 | 2249.04 | 20.53 | 3.13E-02 | -1.4 | GKNGDDGEAGKpGRpGERGPpGP |  | Collagen alpha-1 (I) chain | gi124056487 |
| 87272 | 2319.07 | 33.82 | 4.79E-02 | -1.4 | AGpPGEAGKpGEQGVpGDLGApGPSG |  | Collagen alpha-1 (I) chain | gi124056487 |
| 87460 | 2323.04 | 22.36 | 2.05E-07 | -1.6 | GQNGEpGGKGERGApGEKGEGGPpG |  | Collagen alpha-1 (III) chain | gi124056490 |
| 90344 | 2377.10 | 20.80 | 7.36E-03 | -1.8 | GKNGDDGEAGKpGRpGERGPpGPQ |  | Collagen alpha-1 (I) chain | gi124056487 |
| 93417 | 2446.09 | 28.37 | 2.80E-12 | -2.4 | ADGQpGAKGEpGDAGAKGDAGpPGPAGP |  | Collagen alpha-1 (I) chain | gi124056487 |
| 94308 | 2471.16 | 34.77 | 1.07E-11 | -2.0 | TGPIGPpGPAGApGDKGESGPSGPAGPTG |  | Collagen alpha-1 (I) chain | gi124056487 |
| 94807 | 2483.12 | 27.57 | 7.74E-10 | -2.1 | AGQDGRpGPpGppGARGQAGVmGFpG |  | Collagen alpha-1 (I) chain | gi124056487 |
| 98089 | 2559.18 | 19.41 | 1.79E-19 | 10.5 | DEAGSEADHEGTHSTKRGHAKSRP |  | Fibrinogen alpha chain | gi1706799 |
| 99736 | 2583.15 | 23.68 | 1.98E-09 | -1.6 | ERGEAGIpGVpGAKGEDGKDGSpGEpG |  | Collagen alpha-1 (III) chain | gi124056490 |
| 99808 | 2584.23 | 35.18 | 5.16E-07 | -1.5 | LTGPIGPPGpAGApGDKGESGPSGPAGPTG |  | Collagen alpha-1 (I) chain | gi124056487 |
| 114823 | 2926.30 | 22.22 | 1.26E-07 | -2.3 | ESGREGAPGAEGSpGRDGSpGAKGDRGETGP |  | Collagen alpha-1 (I) chain | gi124056487 |
| 118163 | 3011.39 | 29.75 | 9.77E-15 | -1.7 | LTGSpGSpGpDGKTGPPGPAGQDGRPGPpGppG |  | Collagen alpha-1 (I) chain | gi124056487 |
| 120423 | 3064.32 | 20.57 | 9.80E-09 | -3.0 | EAGRDGNpGNDGPpGRDGQpGHkGERGYPG |  | Collagen alpha-2 (I) chain | gi124056488 |
| 122400 | 3108.45 | 31.28 | 1.29E-06 | -1.5 | ADGQpGAKGEpGDAGAKGDAGpPGPAGPAGPPGpIG |  | Collagen alpha-1 (I) chain | gi124056487 |
| 123671 | 3149.46 | 31.25 | 7.19E-09 | -2.3 | GADGQPGAKGEpGDAGAKGDAGPpGPAGpAGPPGPIG |  | Collagen alpha-1 (I) chain | gi124056487 |
| 124886 | 3193.38 | 22.64 | 5.11E-15 | -2.7 | PpGESGREGAPGAEGSpGRDGSpGAKGDRGETGP |  | Collagen alpha-1 (I) chain | gi124056487 |
| 132383 | 3405.48 | 25.97 | 2.10E-13 | -2.4 | ARGNDGARGSDGQPGPpGppGTAGFpGSpGAKGEVGP |  | Collagen alpha-1 (III) chain | gi124056490 |
| 156081 | 4289.93 | 28.78 | 3.48E-05 | -1.5 | ARGNDGARGSDGQpGppGPPGTAGFPGSpGAKGEVGpAGSpGSNGApG |  | Collagen alpha-1 (III) chain | gi124056490 |

56 identified and validated marker for diabetes. Shown are the protein/peptide identification number in the dataset (Protein ID), mass (in Da) and normalized migration time (in min), the *P*-values [unadjusted using Mann-Withney U-test], regulation factor (F) by diabetes compared to healthy controls [for mean(DM)>mean(control): mean(DM) / mean(control); for mean(DM)<mean(control): -mean(control) / mean(DM)]. In addition, sequences (modified amino acids: p=hydroxyproline; k= hydroxylysine; m=oxidized methionine), protein names, and accession numbers are given.
